# Antibiotic treatment targeting gram negative bacteria prevents neratinib-induced diarrhea in rats

**DOI:** 10.1016/j.neo.2022.100806

**Published:** 2022-05-10

**Authors:** Kate R. Secombe, Imogen A. Ball, Anthony D. Wignall, Emma Bateman, Dorothy M. Keefe, Joanne M. Bowen

**Affiliations:** a School of Biomedicine, University of Adelaide, Adelaide, South Australia, Australia; b Division of Health Sciences, University of South Australia, South Australia, Australia

**Keywords:** Tyrosine kinase inhibitors, Diarrhea, Breast cancer, Rat model, Neratinib

## Abstract

**Background:**

Neratinib is a pan-ErbB tyrosine kinase inhibitor used for extended adjuvant treatment of HER2-positive breast cancer. Diarrhea is the main adverse event associated with neratinib treatment. We aimed here to determine whether antibiotic-induced gut microbial shifts altered development of neratinib-induced diarrhea.

**Methods:**

Female Albino Wistar rats (total *n* = 44) were given antibiotics (vancomycin, neomycin, or a cocktail of vancomycin, neomycin and ampicillin) in drinking water for four weeks, and then treated daily with neratinib (50 mg/kg) for 28 days. Diarrhea, along with markers of gastrointestinal damage and microbial alterations were measured by histopathology and 16S sequencing, respectively.

**Results:**

Rats treated with vancomycin or neomycin had significantly lower levels of diarrhea than rats treated with neratinib alone. In the distal ileum, neratinib was associated with a statistically significant increase in histological damage in all treatment groups expect the antibiotic cocktail. Key features included villous blunting and fusion and some inflammatory infiltrate. Differences in microbial composition at necropsy in vehicle control, neratinib and neratinib + neomycin groups, were characterized by a neratinib-induced increase in gram-negative bacteria that was reversed by neomycin. Neomycin shifted bacterial composition so that *Blautia* become the dominant genus.

**Conclusions:**

Narrow spectrum antibiotics reduced neratinib-induced diarrhea. This suggests that the microbiome may play a key role in the development and prolongation of diarrhea following neratinib treatment, although further research is required to understand the key bacteria and mechanisms by which they reduce diarrhea, as well as how this may impact presentation of diarrhea in clinical cohorts.

## Introduction

Neratinib is an orally available, irreversible small molecule pan-ErbB tyrosine kinase inhibitor (TKI). It is FDA approved for the extended adjuvant treatment of early-stage HER2 positive breast cancer, and in combination with capecitabine for advanced and metastatic HER2 positive breast cancer [1,2]. However, diarrhea has been a major adverse event in clinical trials conducted thus far. Two recent meta-analyses looking at the safety and efficacy profile of neratinib [3] or the risk of gastrointestinal events during neratinib treatment [4] found that most frequently occurring adverse event (all-grade) in neratinib monotherapy was diarrhea. The study by Tao et al. [3] found that diarrhea occurred in 83.9% of patients, while Chen et al. [4] concluded that all-grade diarrhea occurred in 78% of patients.

The phase III ExteNET trial of 2840 patients recruited patients to receive one year of neratinib treatment following one year of trastuzumab. In this trial, without diarrhea prophylaxis, 40% of patients developed severe, grade 3-4 diarrhea [5]. Pre-clinical investigations suggested neratinib-induced diarrhea may be reduced by the corticosteroid budesonide, or the bile acid sequestrant colesevelam [2]. The phase II CONTROL study aimed to replicate this in a clinical setting [6,7]. Grade 3 diarrhea rates were lower than in the ExteNET trial, ranging from 32% of patients in the bile acid sequestrant colestipol + as needed loperamide group, to 15% in a neratinib dose escalation group. No grade 4 diarrhea occurred throughout the trial. Despite this improvement, there remains a gap in finding the most effective way to mitigate neratinib-induced diarrhea.

Previous work has shown a role for the gut microbiome in cancer treatment-related diarrhea. In models of chemotherapy-induced diarrhea, shifts in gut microbial composition are evident and believed to be associated with the pathogenesis of intestinal changes [8,9]. A recent hypothesis suggests that the microbiome may have a similar role in diarrhea from TKI treatments [10,11]. Notably, we have recently shown that neratinib treatment in rats leads to, in the cecal microbiome, changes in abundance of the family *Ruminococcaceae* and the genera *Blautia* and *Oscillospira,* and altered Principal Component clustering between vehicle control and neratinib treated rats [12]. Further investigation is required to understand whether these changes are a key factor in diarrhea development or a downstream effect of other mechanisms. Whilst some emerging evidence suggests a regulatory role for the microbiome in response to immunotherapy [13,14], the mechanisms of how EGFR-targeted therapies affect the microbiome is unknown, and whether there is any relationship to outcomes of therapy. Previous research has demonstrated that antibiotic use may negatively affect efficacy of EGFR-targeted treatment for non-small cell lung cancer [15], but the effect of antibiotics on diarrhea from treatment has not been adequately researched.

As such, this study aimed to determine the impact of various antibiotic therapies on gut microbial changes following neratinib and the effect on neratinib-induced diarrhea. Vancomycin is a glycopeptide antibiotic that is predominantly active against gram-positive bacteria [16]. It is commonly used in patients with *Clostridioides difficile* infection or in multi drug-resistant *Staphylococcus aureus* infections [16]. Neomycin is an aminoglycoside antibiotic that works by causing irreversible binding of nuclear 30S ribosomal subunit [17]. It is most effective against gram-negative organisms, and is used to sterilize the gut before digestive tract surgery [17]. Both are poorly systemically absorbed from the intestinal tract. Finally, we tested a broad-spectrum antibiotic cocktail of vancomycin, neomycin and ampicillin (previously used in a variety of pre-clinical models [18]) that aimed to ablate the gut microbiota.

## Methods

### Chemicals and reagents

Neratinib was kindly provided by Puma Biotechnology. Neratinib was diluted in 0.5% (w/w) hydroxypropyl methylcellulose (HPMC) buffer (Sigma-Aldrich).

### Animals and ethics

All experiments were conducted on female Albino Wistar (AW) rats obtained from the Animal Resource Centre, Perth, Australia. Rats were housed in groups of between 4 and 5 in individually ventilated cages. Temperature was maintained between 19 to 23°C and relative humidity within the range of 45% to 65%; with a 12 hour light/dark cycle. Food and water were consumed *ad libitum*. If rats were experiencing moderate to severe treatment-related toxicity (e.g. diarrhea, weight loss, stress marks) they were allowed soaked chow (normal feed softened in water to ease mastication). Rats were acclimatized to local housing conditions for a minimum of 7 days prior to the first day of dosing. On Day 1 of treatment, the rats were between 7 - 9 weeks old. This study was approved by the Animal Ethics Committee of the University of Adelaide (study number M-2019-025), and complied with the National Health and Medical Research Council (Australia) Code of Practice for Animal Care in Research and Training (2013).

### Antibiotics

Antibiotics were added to sterile drinking water four weeks prior to the beginning of neratinib treatment. Vancomycin hydrochloride was diluted to 0.5 g/L (Cayman Chemicals, #15327) and neomycin trisulfate salt hydrate was diluted to 1 g/L (Sigma-Aldrich, #N1876). The antibiotic cocktail consisted of vancomycin and neomycin as above, in addition to ampicillin sodium salt (1 g/L, Sigma-Aldrich, #A9518). A maltodextrin and 1.2% sucralose mixture (Splenda®) was also added in the antibiotic cocktail at 0.75 g/L to ensure water consumption. Antibiotics were shielded from light and refreshed daily.

### Experimental design

Rats were randomly assigned to study groups as follows: vehicle control (0.5% (w/w) HPMC buffer) and no antibiotics (*n =* 8), neratinib (50 mg/kg) and no antibiotics (*n =* 10), neratinib (50 mg/kg) and antibiotic cocktail (*n =* 8), neratinib (50 mg/kg) and vancomycin (*n =* 8) and neratinib and neomycin (*n =* 10). During the 28 day neratinib treatment period, rats received daily oral gavages using a soft plastic feeding tube coated in 30% sucrose solution. Neratinib or vehicle controls were given at a constant dose volume of approximately 5 mL/kg. Individual dose volumes were adjusted daily according to the body weight of each rat on each treatment day. The first day of dosing was designated Day 1. The final dose was given on the day before scheduled necropsy. All rats were deeply anaesthetized via isoflurane inhalation, and culled by cardiac exsanguination with death confirmed by cervical dislocation.

### Clinical gut toxicity assessment

Rats were weighed once daily, and comprehensively monitored twice daily via a clinical symptom reporting system. Diarrhea was graded by two assessors (KRS and IAB) according to a well-established grading system [19] with four grades: 0, no diarrhea; 1, mild (soft unformed stools); 2, moderate (perianal staining and loose stools); and 3, severe (watery stools and staining over legs and abdomen). Rats were to be euthanized if displaying 15% or greater weight loss from baseline or significant distress and clinical deterioration (although no animals reached this endpoint).

### Tissue collection and preparation

At necropsy, the gastrointestinal tract was removed from the pyloric sphincter to the rectum. The small and large intestine were flushed with chilled, sterile 1 x phosphate buffered saline (PBS) and weighed. Samples of duodenum, jejunum, proximal and distal ileum and proximal and distal colon were collected and fixed in 10% formalin for embedding in paraffin.

### Histological examination

Paraffin embedded intestinal samples were cut with a rotary microtome (RM2235, Leica) and 4 µm sections were mounted onto Superfrost glass slides (Menzel-Glaser). Images of all slides were taken using a Nanozoomer digital slide scanner (Hamamatsu Photonics, Japan) and viewed using the Nanozoomer Digital Pathology Software (NDP View v1.2, Histalim). All analysis was conducted in a blinded fashion.

### Mucosal damage analysis

Routine hematoxylin and eosin (H&E) staining was completed and an injury score assigned using a well-established system of histological criteria [20,21]. Criteria were villus fusion, villus atrophy, disruption of brush border and surface enterocytes, crypt losses/architectural disruption, disruption of crypt cells, infiltration of polymorphonuclear cells and lymphocytes, dilation of lymphatics and capillaries and edema. The latter six criteria were examined in the colon. Each criterion was scored as present = 1 or absent = 0.

### Serum endotoxin analysis

Blood samples were collected at necropsy by cardiac puncture using a 23 gauge needle in Z Serum (Sep) Clot Activator Vacuette tubes (Greiner Bio-One). Serum was separated by centrifugation at 931 *x g* for 15 minutes at room temperature. Serum was then aliquoted and stored at -80°C until used.

A serum limulus amebocyte lysate (LAL) endotoxin assay was run on heat-treated serum samples. Serum was diluted 1:10 in endotoxin free water, and then heat treated at 70°C for 15 minutes. The PyroGene Recombinant Factor C Endotoxin Detection Assay (Lonza; #50-658U) was then used to quantify serum endotoxin, as per manufacturer's guidelines. Endotoxin concentration was determined relative to a linear standard curve (range, 0.005–5 EU/mL).

### Gut bacterial DNA extraction and diversity profiling

Fecal samples from vehicle control, neratinib only, and neratinib + neomycin groups were analyzed using 16S sequencing techniques. Distal colon fecal contents were aseptically collected during dissection into a sterile tube, and stored at -80˚C. Samples were sent to the Australian Genome Research Facility (AGRF) for DNA extraction and 16S ribosomal RNA (rRNA) gene region analysis. DNA was extracted from 250 mg of fecal sample using the Qiagen DNeasy PowerLyzer PowerSoil Kits with the PowerLyzer 24 Homogenizer. 16S analysis sequencing details are as follows:

Target: 16S: 341F (V3-V4) (V1-V3), read length = 300bp.
Forward sequence: 5’ CCTAYGGGRBGCASCAG 3’
Reverse Sequence: 5’ GGACTACNNGGGTATCTAAT 3’

Internal blank controls were used in all stages of the library preparation process. Image analysis was performed in real time by the MiSeq Control Software (MCS) v2.6.2.1 and Real Time Analysis (RTA) v1.18.54, running on the instrument computer. The Illumina bcl2fastq 2.20.0.422 pipeline was used to generate the sequence data. CLC Genomics Workbench 12.0 (https://www.qiagenbioinformatics.com/) was used to complete bacterial diversity profiling. Paired-ends reads were assembled by aligning forward and reverse reads. Primers were identified and trimmed. Trimmed sequences were quality filtered, duplicate sequences removed and sorted by abundance. Reads were assigned to taxonomic identities using the Greengenes 97% similarity database version 13.8. Alpha-diversity was calculated using the Shannon diversity index. Beta diversity of operational taxonomic units (OTUs) was calculated using Principal Coordinate Analysis (PCoA) based on generalized UniFrac distances [22]. The program BURRITO [23] was used to visualize the links between taxonomic composition and function in the dataset using KEGG pathways. Principal Component Analysis (PCA) was used to assess differences in KEGG pathways.

### Statistical analysis

Data were compared using Prism version 7.0 (GraphPad Software, USA). If data was normally distributed, bars on graphs are mean ± SEM. If not, median is displayed in graphs. The assumptions of equality of variance for each group and normally distributed data were tested using Bartlett's test and D'Agostino & Pearson omnibus normality test, respectively. If these assumptions were violated, non-parametric equivalent tests were performed, including Kruskal-Wallis for independent data. When assumptions held, ANOVA's were performed using the 2-way analysis of variance (ANOVA). A Mantel-Cox test was used to determine differences in the survival curves for diarrhea levels. Statistical Analysis of Metagenomic Profiles (STAMP) [24] was used to assess the predicted metagenome using Welch's t test with the Benjamini-Hochberg correction for the false-discovery rate (FDR). P-values less than 0.05 were considered statistically significant.

## Results

### Single narrow-spectrum antibiotics significantly reduced diarrhea levels

**Fig. 1:**
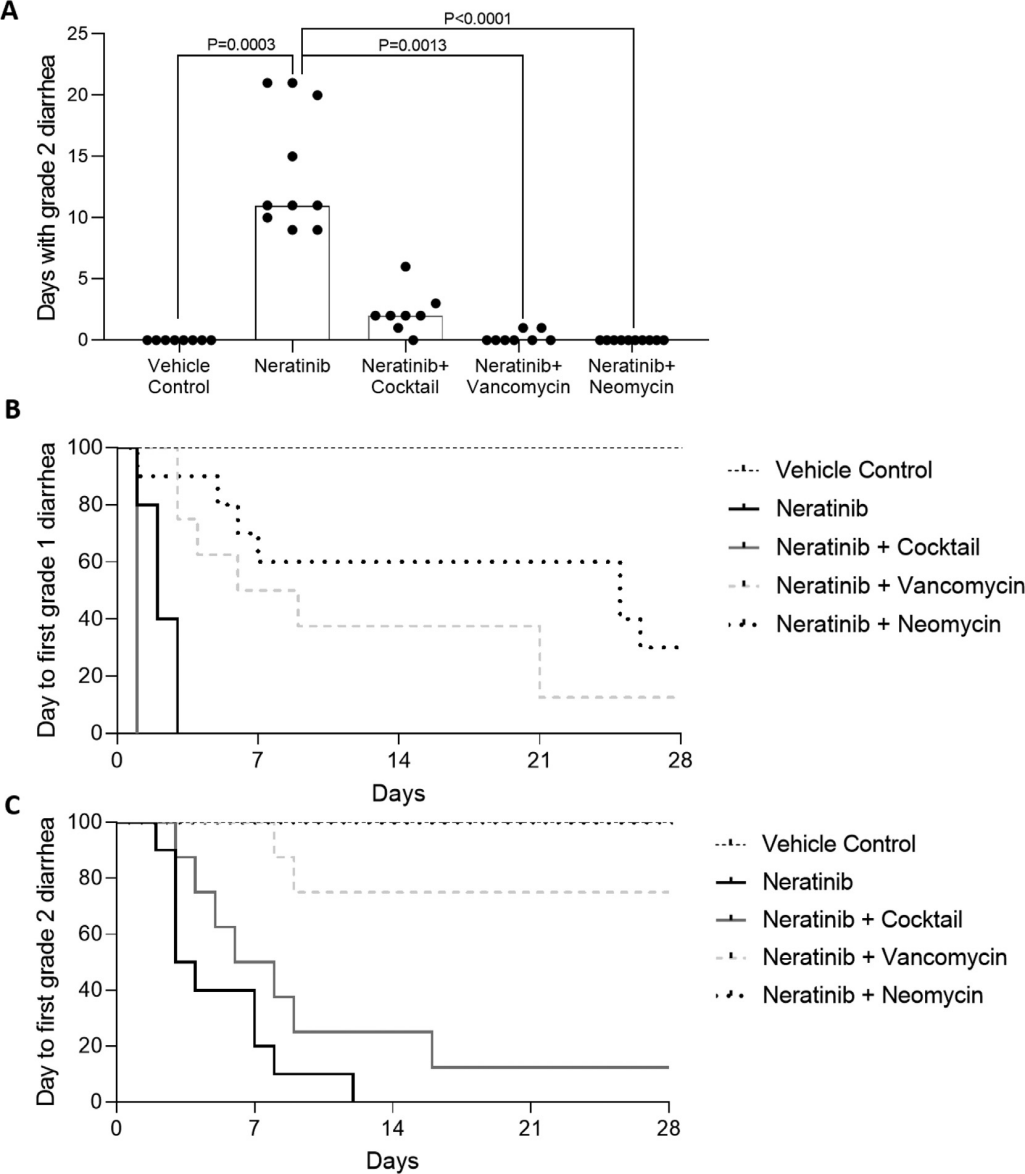
Diarrhea development. A) Days in total with grade 2 (moderate) diarrhea. Bar signifies median. Rats treated with vancomycin (*P =* 0.0013) or neomycin (*P <* 0.0001) had significantly less days with diarrhea compared to the neratinib alone group. Kruskal-Wallis test used to determine significance. B) Survival graph of days until first occurrence of grade 1 diarrhea. C) Survival graph of days until first occurrence of grade 2 diarrhea. Antibiotic treated groups had significantly different survival curves to neratinib alone in both (A) and (B) (Mantel-Cox test, *P <* 0.0001).

Neratinib induced significantly longer duration of moderate (grade 2) diarrhea (mea*n =* 13.8 days, range=9-21) compared to the vehicle control group (mea*n =* 0 days, range=0-0, *P* = 0.0003) (Fig. 1a). The addition of vancomycin (mea*n =* 0.25, range=0-1 days, *P =* 0.0013) and neomycin (mea*n =* 0 days, range=0-0, *P* < 0.0001) significantly reduced days with grade 2 neratinib-induced diarrhea compared to neratinib alone. There was no significant difference between grade 2 diarrhea in the antibiotic cocktail (mea*n =* 2.25 days, range=0-6, *P* > 0.05) and neratinib alone. The antibiotic cocktail caused mild grade 1 diarrhea which preceded the beginning of neratinib treatment (Fig. 1b). Only two rats developed grade 2 or 3 diarrhea in the vancomycin group, and no animal in the neomycin group developed grade 2 or 3 diarrhea (Fig. 1c).

### Rats treated with antibiotic cocktail gained less weight than all other groups

**Fig. 2:**
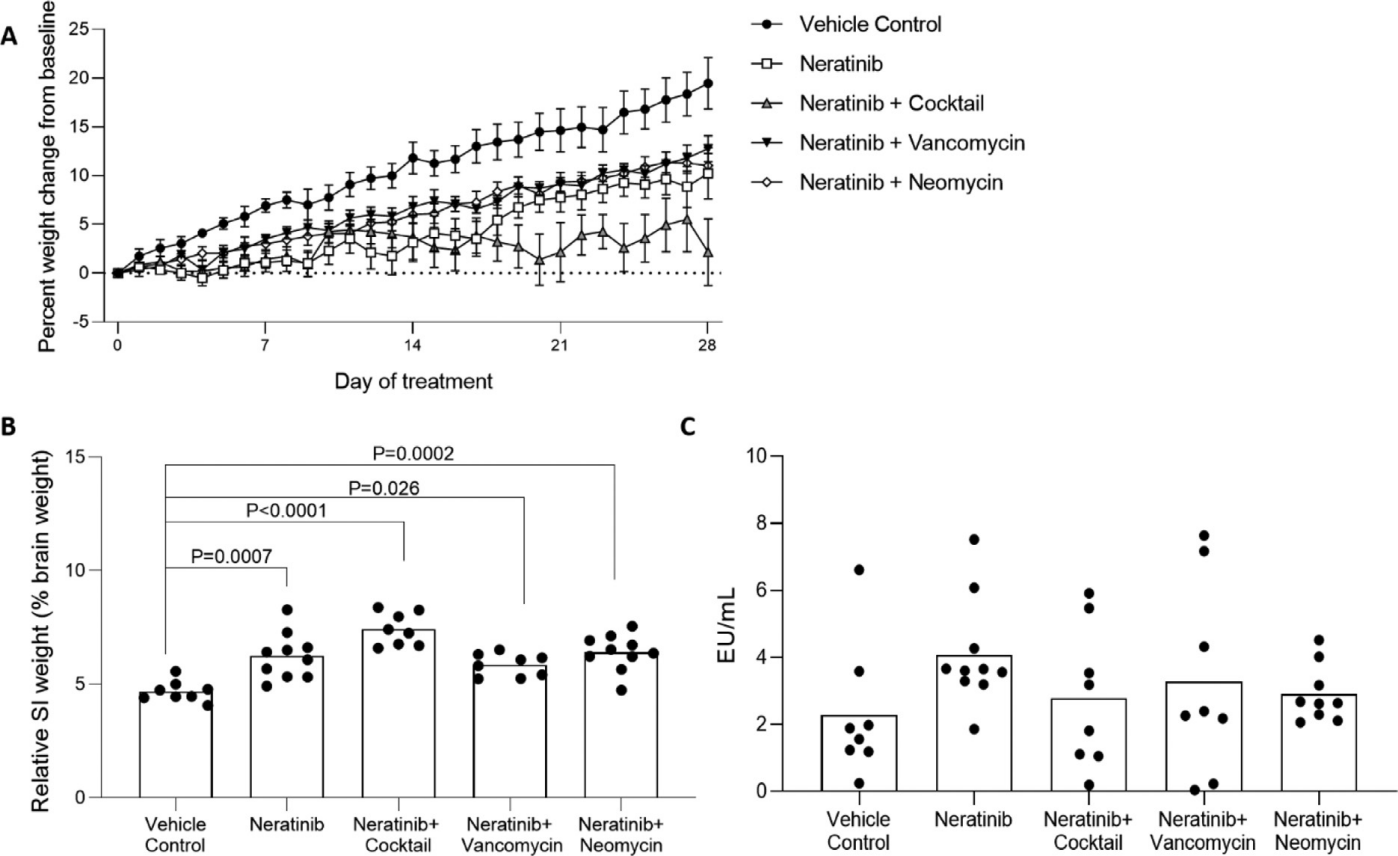
Body, organ weight and serum endotoxin investigations. A) Baseline-corrected weight gain across 28 days of treatment. Rats treated with antibiotic cocktail had significantly less weight gain than all other groups of rats (*P <* 0.0001). There was no significant difference between the vehicle control group and the neratinib + vancomycin or neratinib + neomycin groups (*P >* 0.05). Data shown as mean±SEM. Kruskal-Wallis test used. B) Small intestinal weight normalized to brain weight. All neratinib treated groups increased significantly compared to vehicle control (one-way ANOVA with Tukey's multiple comparison test). C) Serum endotoxin was assessed using a serum Limulus Amebocyte Lysate (LAL) endotoxin assay. Bar signifies mean. Samples were analysed using a one-way ANOVA with Tukey post-hoc testing. There were no significant differences between groups (*P >* 0.05). One outlier was removed from the neratinib + neomycin group as it was more than 3 standard deviations above the mean, due to a haemolysed sample. Bars signify mean.

All rats continuously gained weight over the course of the experiment. Neratinib only, and neratinib + antibiotic cocktail treated rats gained significantly less weight over the time course than vehicle controls rats (*P <* 0.0001). The neratinib and cocktail group also gained significantly less weight than the vancomycin group (*P =* 0.0082) and the neomycin group (*P =* 0.014) (Fig. 2a).

### Neratinib increased small intestinal weight in all groups

The wet weight of the small intestine was normalized to percentage of brain weight. All neratinib treated groups significantly increased compared to vehicle control (mea*n =* 4.69%) (Fig. 2b). The neratinib + antibiotic cocktail group had the highest mean (7.42%, *P <* 0.0001 compared to control). The neratinib only group had a mean of 6.24% (*P =* 0.0007), neratinib + vancomycin had a mean of 5.85% (*P =* 0.026) and the neomycin group had a mean of 6.41% (*P =* 0.0002). There were no significant differences in weight of the large intestine, liver, spleen, kidneys, stomach, heart or lungs (data not shown).

### No change in serum endotoxin between groups

Endotoxin assay results were variable within groups, and there were no significant differences between groups (Fig. 2c). Values were within previously published ranges of healthy female Wistar rats [25].

### Neratinib caused significant intestinal injury in the ileum

**Fig. 3:**
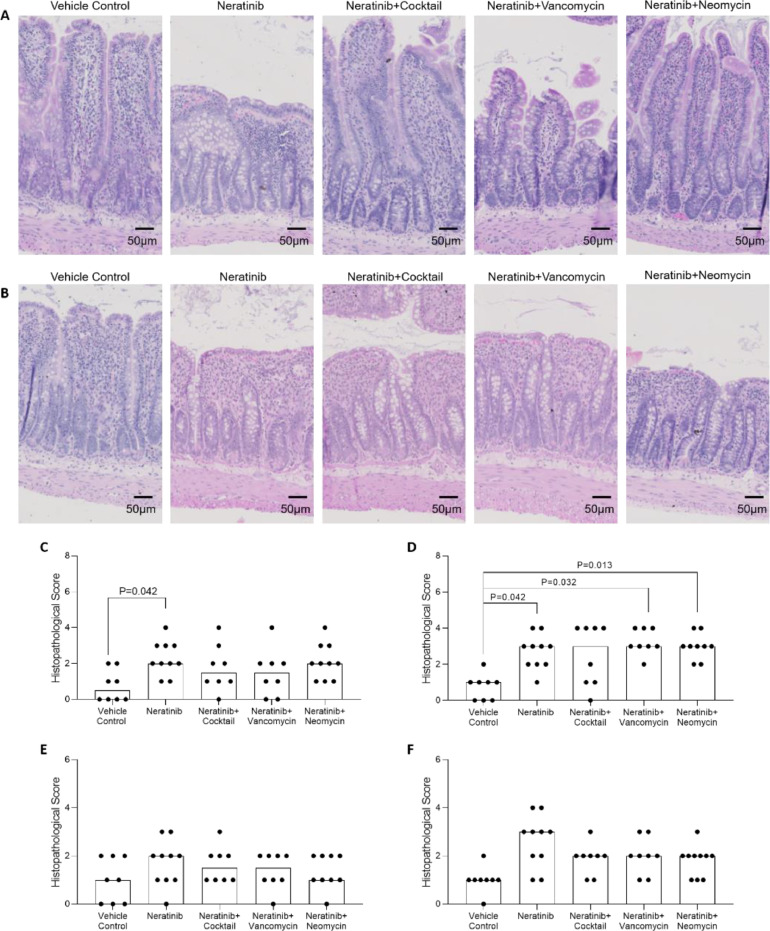
Intestinal histopathological injury. Representative images of hematoxylin and eosin staining in the proximal (A) and distal (B) ileum: original magnification is 200×; scale bars represent 50 μm as shown in images. Histological damage scoring in the proximal ileum (C), distal ileum (D), proximal colon (E) and distal colon (F). Bar signifies median. In the distal ileum, neratinib alone injury scores were significantly higher than vehicle control (*P =* 0.042). In the proximal ileum, neratinib alone (*P =* 0.042), neratinib + vancomycin (*P =* 0.032) and neratinib + neomycin (*P =* 0.013) had significantly higher scores than vehicle control. There were no significant differences in the colon. Statistical significance determined using a Kruskal–Wallis test. Damage scoring on a scale of 0-8 for C and D and 0-6 for E and F.

In the proximal ileum (Fig. 3a, c), neratinib caused significantly increased histopathological injury compared to vehicle control (*P =* 0.042). Antibiotic treated groups were not increased compared to vehicle control (*P >* 0.05). Blunting and fusion of villi was observed in the neratinib only group. In the distal ileum (Fig. 3b, d), neratinib only and single antibiotic treated groups had significantly higher levels of damage than vehicle control (control vs neratinib; *P =* 0.042, control vs vancomycin, *P =* 0.032, control vs neomycin, *P =* 0.013). In the distal ileum, blunting and fusion of the villi was observed in all neratinib treated groups. No significant differences were observed between groups in the distal or proximal colon (Fig. 3e, f).

### Microbial changes

**Fig. 4:**
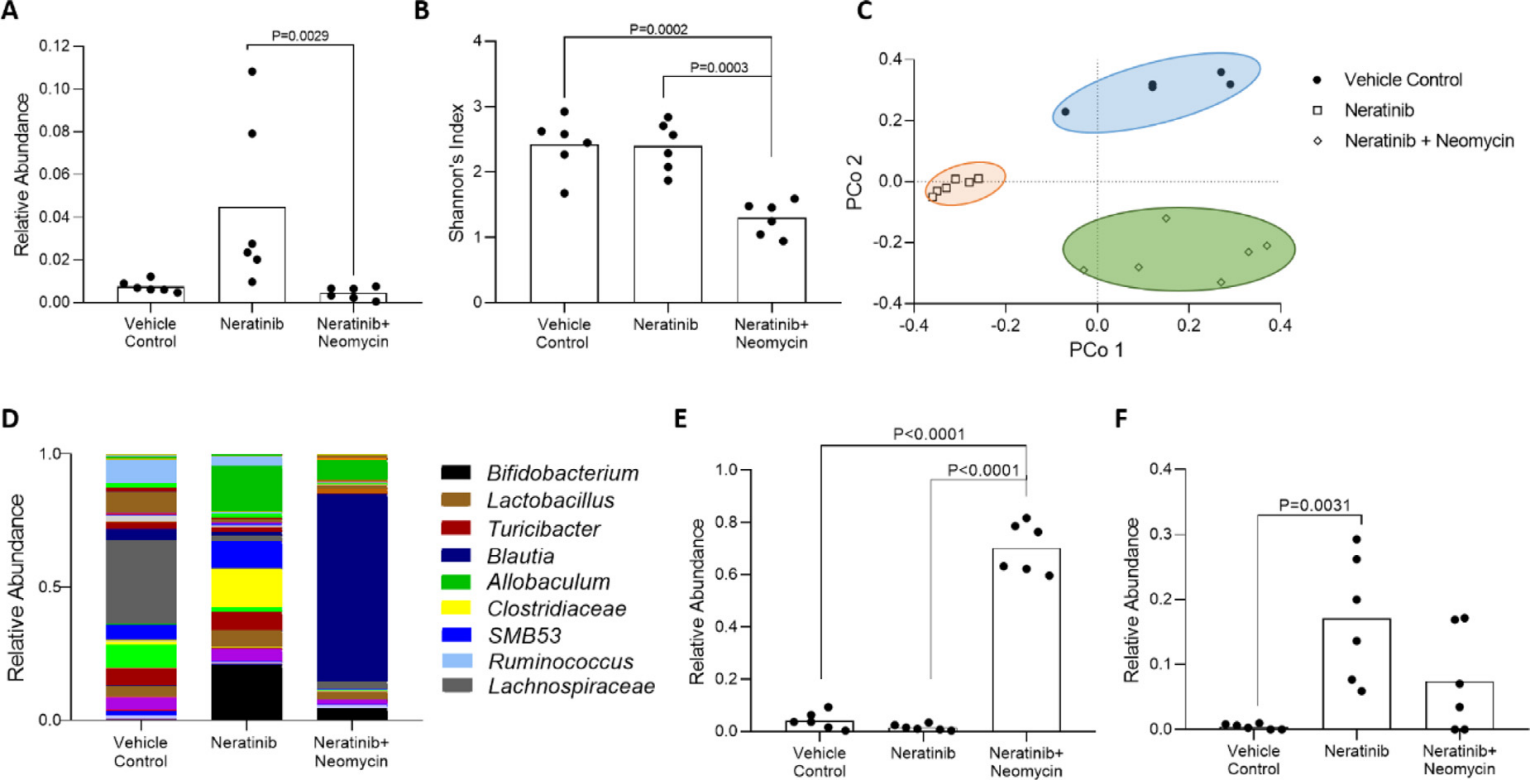
Microbial analysis. (A) Relative abundance of the gram-negative phylum Proteobacteria. The neratinib group had significantly higher levels compared to neratinib + neomycin (*P =* 0.0029). (B) Alpha diversity measured using Shannon's index. Neomycin treated rats had significantly lower diversity than both vehicle control (*P =* 0.0002) and neratinib only (*P =* 0.0003). (C) Principal coordinate analysis demonstrated that each group clustered separately, confirmed by PERMANOVA (*P =* 0.0065). (D) Relative abundance at the genus level. Highly abundant genera shown in figure legend. (E) Relative abundance of *Blautia*. The neratinib and neomycin group had significantly higher levels than vehicle control (*P <* 0.0001) and neratinib only (*P <* 0.0001). (F) Relative abundance of *Allobaculum*. Neratinib only had significantly higher levels than vehicle control (*P =* 0.0031). No significant difference was seen in the neomycin treated group. In (B), (C), (D) and (E), bar signifies mean and significance determined via 2-way ANOVA or Kruskal-Wallis test.

To assess the fecal microbiome, distal colonic pellets were taken at necropsy from a subset of 6 rats from each of the vehicle control, neratinib only and neratinib + neomycin groups. The neomycin group was the focus of microbial analysis since these rats experienced no grade 2 diarrhea. Samples were analyzed using 16S sequencing (Fig. 4). To ensure the targeting effect of neomycin on gram-negative bacteria, we analyzed relative abundance of the phylum Proteobacteria. Proteobacteria are gram-negative, and are often present at low levels in healthy controls but increased in instances of inflammation or dysbiosis [26]. Here, neratinib alone treated animals had significantly higher levels of Proteobacteria than neratinib + neomycin treated rats (*P =* 0.0029), whereas there was no significant difference between vehicle control rats and neratinib + neomycin treated rats (*P >* 0.05) (Fig. 4a). At species level, the neomycin treated group had significantly lowered alpha diversity (Shannon's index) than both the vehicle control group (*P =* 0.0002) and the neratinib alone group (*P =* 0.003) as expected (Fig. 4b). There were no significant differences between the vehicle control and neratinib groups. The same pattern was seen at the genus level. Principal coordinate analysis showed that each group clustered differently, confirmed by pairwise PERMANOVA tests (*P =* 0.0065, Fig. 4c).

At genus level, there were marked differences between each group in the fecal microbiome at necropsy (Fig. 4d). There was a significant increase in the relative abundance of the genus *Blautia* in the neratinib + neomycin group (*P <* 0.001) compared to both vehicle control and neratinib only (Fig. 4e). The genus *Allobaculum* was also significantly increased in the neratinib group compared to vehicle control (*P =* 0.0031). This significant difference was not seen in the neratinib + neomycin group (Fig. 4f).

**Fig. 5:**
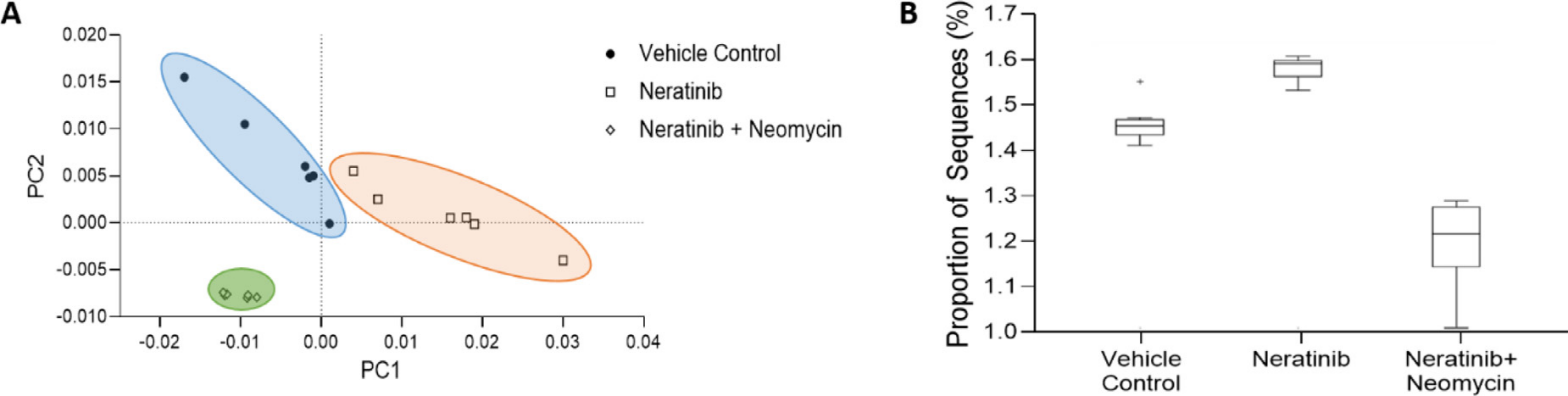
Inferred gut microbiome functions analyzed using STAMP from 16S rRNA gene sequences. (A) PCA plot at the KEGG level showing separate clustering between each treatment group. (B) Alterations in oxidative phosphorylation function between groups. Box plots show the top quartile, median and bottom quartile, with ‘+’ indicating outliers. Benjamini–Hochberg FDR method was used to correct multiple comparisons.

Finally, analysis of metabolic pathways predicted to be altered due to microbial changes were investigated. The Nearest Sequenced Taxon Index (NSTI) scores of each sample varied from 0.073 to 0.17 (mean 0.11, data not shown). These scores are moderate, suggesting somewhat accurate and reliable predictions [27]. However without functional tests of the metabolome, it would not be prudent to completely rely on these findings. At the KEGG pathway level, results were filtered to those with a corrected p-value of <0.01 and an effect size of >0.85. The four functional groups with the highest effect size between treatment groups were all related to metabolism (methane metabolism, lysine biosynthesis, glyoxylate and dicarboxylate metabolism and cysteine and methionine metabolism) (Table 1). Principal component analysis (PCA) demonstrates altered clustering between the treatment groups (Fig. 5a). Also of particular note was levels of oxidative phosphorylation, levels of which were significantly lower in the neratinib alone treated group compared to vehicle control and neratinib + neomycin groups (Fig. 5b).

**Table 1:** Analysis of metabolic pathways predicted to be altered due to microbial changes. Results were filtered to the four functional groups with the highest effect size between treatments groups.

KEGG Level			*p*-values (corrected)	Effect size	Mean relative frequency (%)		
1	2	3			Vehicle Control	Neratinib	Neomycin
Metabolism	Energy metabolism	Methane metabolism	1.73×10^−8^	0.94721	0.43589	0.410734	0.738497
Metabolism	Amino acid metabolism	Lysine biosynthesis	1.09×10^−8^	0.945554	0.782927	0.74089	0.929823
Metabolism	Carbohydrate metabolism	Glyoxylate and dicarboxylate metabolism	4.54×10^−8^	0.930514	0.723007	0.674503	0.798065
Metabolism	Amino acid metabolism	Cysteine and methionine metabolism	8.81×10^−8^	0.92111	0.958553	1.148907	0.833396

## Discussion

This study aimed to investigate how alterations in gut microbial composition affect the development of neratinib-induced diarrhea. We found that narrow spectrum antibiotics targeting specifically gram-negative or positive bacteria (neomycin or vancomycin respectively) caused a highly significant decrease in diarrhea levels, whereas a broad spectrum antibiotic cocktail was less effective.

While neomycin and vancomycin treatment significantly reduced diarrhea and caused a weight gain pattern more similar to vehicle controls than neratinib only treatment, antibiotic treatment did not cause improvement in the pathological markers measured in the distal ileum. In addition, no changes in serum endotoxin levels were noted for any group, indicating that the mucosal barrier remained intact. More research may be required to understand the exact way in which neomycin and vancomycin were able to cause such vast improvement in diarrhea levels without modifying gross-histological changes to the tissue. It is unlikely to be due to direct inhibition of neratinib absorption, although serum concentration was not measured in the current study to definitively exclude this concern. One recent review [28] has suggested that antibiotics in the macrolide groups may decrease TKI metabolism via inhibition of CYP3A4, however there is a lack of research showing whether this effect occurs in other antibiotics. Additionally, while there is evidence to suggest that the gut microbiome assists in the metabolism of chemotherapies, there is no clear evidence as of yet to suggest a similar effect in neratinib or other TKIs [29].

Neomycin and vancomycin are minimally absorbed from the gastrointestinal tract when administered orally and therefore is the basis of their use to specifically suppress intestinal bacteria [30]. There is however some evidence to suggest that systemic uptake of vancomycin may be increased in instances of intestinal inflammation [31]. Neomycin is an aminoglycoside antibiotic with strong activity against gram-negative bacteria. We confirmed this, showing that rats treated with neomycin had significantly lower levels of gram-negative Proteobacteria compared to rats treated with only neratinib. Previously, pre-clinical studies have shown extensive increases in pathogenic gram-negative bacteria following various cancer treatments corresponding with an increase in diarrhea [8,[32], [33], [34]]. Additionally, these gram-negative species can often release lipopolysaccharide (LPS) known to initiate the key inflammatory mediators that are known to cause diarrhea following cancer treatment [35]. Furthermore, a small study showed complete amelioration of diarrhea in 6 out of 7 patients receiving the chemotherapy agent irinotecan as well as neomycin [36]. Vancomycin, when orally administered, is commonly used to treat intestinal *Clostridioides difficile* infection. It is highly effective against gram-positive bacteria. As both vancomycin and neomycin had similar effectiveness in decreasing diarrhea following neratinib, this diarrhea does not appear to be due to a gram-positive or negative specific effect.

The antibiotic cocktail used was not as successful in reducing diarrhea compared to the single antibiotics. In fact, mild diarrhea was noted in this group prior to the first neratinib treatment. This may be due to the sucralose artificial sweetener that was added causing further non-beneficial changes to the microbiome [37]. However, the same antibiotic cocktail with sucralose at a higher concentration has been used in our laboratory [38] and by others [39] in Sprague-Dawley rats with no diarrhea noted. Additional research has administered sucralose long-term via gavage or in feed, with no mention of diarrhea or gastrointestinal issues [40,41]. Alternatively, the broad spectrum nature (vancomycin, neomycin and ampicillin) of the antibiotic combination could have led to a form of antibiotic-induced diarrhea, although similarly, no previous research using a similar antibiotic cocktail has reported such instances of diarrhea [18]. Future studies could also perform colony-forming unit assays on culturable bacteria in the stool, to assess the level of microbial ablation in antibiotic treated groups.

A key unknown regarding the microbiome in TKI-induced diarrhea is whether microbiome changes cause diarrhea, or if these changes are simply a consequence of diarrhea occurring via other mechanisms. Many studies, both pre-clinical and clinical, have shown changes to the gut microbiome following treatment with TKIs, or indeed that people with diarrhea had a different microbial composition to those who did not develop diarrhea [11,42]. However, this study is the first to show that alterations in the gut microbiome before treatment begins leads to changes in diarrhea severity. This follows a recent hypothesis by Wardill and Tissing that the pre-treatment gut microbial composition could be used to predict risk of developing gut toxicity from a range of cancer treatments [43]. However, future research may focus on whether the microbiome has a key role in the development of diarrhea, or if it is more likely to be exacerbating and prolonging diarrheal symptoms that are due to other mechanisms such as chloride secretion or epithelial damage [44]. While our results support a causative role for microbiome composition in development of diarrhea, the specific microbes most important have not been identified in this model. Studies of germ-free mice monocolonized with specific bacteria may be a useful model for future studies.

In this study, relative abundance of the *Blautia* genus was highly increased in neomycin treated rats, who had less diarrhea than neratinib alone treated rats. Other studies have similarly shown a relationship between decreases in *Blautia* and incidence of cancer-treatment induced diarrhea [45]. In particular, our previous study showed that vehicle control treated rats had higher levels of *Blautia* compared to neratinib-treated rats [12]. *Blautia* is a genus of obligate anerobic bacteria that is of increasing research interest in gut health. *Blautia* falls within the Lachnospiraceae family, which is important in breaking down polysaccharides consumed in the diet to short chain fatty acids (SCFA) including acetate, butyrate and propionate. Therefore, the presence of *Blautia* may support a healthy microbiome composition, and its presence in the intestine has also been associated with reduced deaths from Graft-versus host disease [46].

In our studies, we have shown that histological damage of the gut stemming from neratinib is mainly focused on the ileum, however here we studied the fecal microbiome. There is known to be high variation in the spatiotemporal organization of the microbiome through the length of the intestinal tract, due to pH and oxygen levels. It is possible that the changes we saw in the fecal microbiome may be different to the changes in the ileal microbiome. In addition to differences through the length of the intestine, further research could begin to assess the differences between the mucosal and luminal microbiomes following neratinib treatment. It has previously been shown that Lachnospiraceae, the family that *Blautia* falls within, is found in high volumes in the transverse folds of the proximal colon. These areas provide a protected area that is separated from the luminal flow of digesta. Subsequently, microbes localized to such safe havens likely have an advantage in recolonizing the intestine after disruption by antibiotics and infection [47]. Additionally, bacterial composition has been shown to affect the structure and function of the intestinal mucus layer [48].

As a relationship has been found between the microbiota and breast cancer development and treatment response [49], a potential limitation of this study was the lack of a tumor-bearing rat model. This study was designed based on the first approved indication of neratinib, in the extended adjuvant setting of early breast cancer, where there will be no tumor remaining post-surgery. Neratinib is now also used in a metastatic breast cancer setting in combination with capecitabine, were these microbiota-tumor interactions may play a more significant role. In future, a tumor-bearing model would be appropriate when studying the combination treatment in this setting.

This study has opened new questions and research avenues in investigating the link between the microbiome and neratinib-induced diarrhea. In particular, this study shows that we may have an opportunity to specifically target specific gut microbiome profiles in order to reduce diarrhea from neratinib and other TKIs. However, a deeper understanding of the exact microbiome changes in patients is required to precisely target the desired microbial profile. One study has previously used network analysis to stratify renal cell carcinoma patients with prior exposure to TKIs and antibiotics using gut microbiome composition [50]. This approach could be broadened to determine microbial profiles that lead to development of toxicity.

## Conclusion

High levels of diarrhea occur in many patients being treated with neratinib, and current treatments both carry risks of side effects, and may not be specific to the pathogenesis of this diarrhea. In this study we have shown that narrow spectrum antibiotics are able to reduce the development of diarrhea. An antibiotic cocktail was not as successful. Overall, these results suggest that the microbiome may play a key role in the development and prolongation of diarrhea following neratinib treatment, although further research is required to understand the exact mechanisms that have reduced diarrhea in this study.

Ethical approval: All applicable international, national, and/or institutional guidelines for the care and use of animals were followed. All procedures performed in studies involving animals were in accordance with the ethical standards of the institution or practice at which the studies were conducted. This article does not contain any studies with human participants performed by any of the authors.

## Funding

This research was funded by Puma Biotechnology.

## CRediT authorship contribution statement

**Kate R. Secombe:** Conceptualization, Methodology, Formal analysis, Investigation, Writing – original draft, Visualization. **Imogen A. Ball:** Methodology, Investigation, Project administration, Writing – review & editing. **Anthony D. Wignall:** Methodology, Investigation, Writing – review & editing. **Emma Bateman:** Methodology, Investigation, Writing – review & editing. **Dorothy M. Keefe:** Funding acquisition, Conceptualization, Writing – review & editing. **Joanne M. Bowen:** Conceptualization, Methodology, Writing – review & editing, Supervision, Project administration, Funding acquisition.
